# Strengthening access to zinc and oral rehydration solution for childhood diarrheal treatment in Africa

**DOI:** 10.1186/s41256-025-00428-8

**Published:** 2025-12-15

**Authors:** Nsikakabasi Samuel George, Sandra Salomy Phiri, Deborah Oluwaseun Shomuyiwa, Martha Mwaba, Francis Ima Imoke, Isack Hokelai Kaniki, Grace Mmesomachukwu Oji, Lucky Iseghehi

**Affiliations:** 1 School of Medicine and Population Health, The University of Sheffield, Sheffield, UK; 2 Institute of Public Health, Faculty of Health Sciences, Jagiellonian University Medical College, 31-066, Krakow, Poland; 3 Youth On Board Organization (YOBO), Lilongwe, Malawi; 4 Faculty of Pharmacy, University of Lagos, Lagos, Nigeria; 5 Ministry of Health, Lusaka, Zambia; 6 Department of Public Health, University of Calabar, Calabar, Nigeria; 7 Dodoma Regional Referral Hospital, Dodoma, Tanzania; 8 eHealth Africa, Kano State, Nigeria

**Keywords:** Diarrhea treatment, Health systems, Child health, Health promotion, Health systems strengthening

## Abstract

Childhood diarrhea remains a formidable public health challenge in Africa, exacerbated by limited access to clean water, poor sanitation, and inadequate healthcare infrastructure, impacting millions of children annually. In sub-Saharan Africa, diarrhea ranks as the leading cause of death among children under five, representing 9% of all deaths in this age group. Zinc supplementation and oral rehydration solution have emerged as pivotal treatments for childhood diarrhea, addressing electrolyte imbalances, dehydration, reducing the severity and duration of diarrhea episodes, while rehydrating children by replacing lost fluids and electrolytes. However, there are several challenges in accessing these essential components, including limited availability, affordability, supply chain challenges, and lack of awareness. In this article, we explored the burden of diarrheal diseases, the impact of zinc and oral rehydration solution, and highlight the urgent need to prioritize and strengthen access to these interventions to significantly improve treatment outcomes and reduce the burden of childhood disease in Africa. Through implementing key strategies such as addressing affordability of these commodities, training and capacity building and supply chain strengthening, community engagement, and monitoring and evaluation, significant progress can be made in overcoming barriers and improving health outcomes for children. These will ultimately contribute to the reduction of childhood mortality and the attainment of global health goals in Africa.

## Introduction

Childhood diarrhea remains a formidable public health challenge in Africa, exacerbated by limited access to clean water, poor sanitation, and inadequate healthcare infrastructure, impacting millions of children annually [1, 2]. With diarrheal diseases accounting for over 440,000 deaths annually among children under five in Africa, representing 9% of all deaths in this age group, the urgency of addressing this issue cannot be overstated [1, 2]. Sub-Saharan Africa bears a disproportionate burden, with diarrhea ranking as the leading cause of death among children under five, further straining healthcare systems in the region [1].

Zinc supplementation and oral rehydration solution (ORS) have emerged as pivotal treatments for childhood diarrhea, as recommended by the World Health Organization (WHO) and the United Nations Children’s Fund (UNICEF) [3]. Zinc’s documented role in reducing the severity and duration of diarrhea episodes, coupled with ORS addressing electrolyte imbalances and dehydration, present promising avenues for treatment [3–6]. However, despite their effectiveness, access to zinc and ORS remains a challenge in sub-Saharan Africa, with low utilization rates reported in countries like Nigeria, Burundi, and Kenya [7]. Research from Sudan and Ethiopia reveals significant gaps in treatment coverage, indicating the urgent need to improve access to these life-saving interventions [4, 5]. This paper will explore the burden of diarrheal diseases, impact of zinc and ORS in managing childhood diarrhea, highlighting the urgent need to prioritize and strengthen access to these interventions to significantly improve treatment outcomes and reduce the burden of childhood diarrhea in the region. 

## Burden of childhood diarrhea in Africa

Diarrhea is a leading cause of death among children under five in Africa, with a weighted prevalence of about 15% across African countries [1, 2, 8, 9]. Across sub-Saharan African countries, diarrhea is ranked the 5th leading cause of disability-adjusted life year (DALYs), according to the 2021 Global Burden of Disease (GBD) study by the Institute for Health Metrics and Evaluation (IHME) [10]. These rates range from 162.33 DALYs per 100,000 population in Seychelles to 11,662.54 DALYs per 100,000 population in Chad. The case fatality rate for childhood diarrhea in Africa varies from 2% to 9%, depending on healthcare access, nutritional status, and comorbidities [10]. Taking a gender distribution, DALYs are higher among males than females across all countries. However, it is worthy to note that diarrhea-related mortality rates in Africa have reduced consistently in the last 20 years [9, 10]. In African countries currently experiencing crisis and conflicts including but not limited to Mali, Sudan, DR Congo, Somaliland, Northern Nigeria and Burkina Faso, diarrheal diseases are more prevalent among displaced children, and in refugee and internally displaced people camps [11].

Several factors contribute to the persistent high incidence and prevalence of diarrhea [10, 12]. Inadequate access to clean water, sanitation facilities, and proper hygiene practices increase the risk of diarrheal illnesses. Malnutrition in all forms including wasting, stunting and underweight increases the risk of diarrheal diseases. Furthermore, the rise of antimicrobial resistance (AMR) in childhood contributes significantly to the burden of childhood diarrhea in Africa [13, 14]. Although diarrhea requires supportive care rather than antibiotics, antibiotics are frequently prescribed, primarily due to ambiguities, the urgency for immediate alleviation, and a lack of awareness among healthcare providers [13, 15, 16]. This practice not only neglects the underlying cause but also fosters the development of resistant bacterial strains, creating a vicious cycle where children face repeated exposure to resistant infections that become increasingly difficult to treat effectively [13, 15]. The Global Burden of Disease (GBD) study reported that in 2019, bacterial AMR was associated with an estimated 1.05 million deaths, including 250,000 deaths within the WHO African region [17]. Among these, diarrheal diseases significantly contributed to the burden of AMR in the region. Specifically, diarrheal diseases accounted for an estimated 27,000 deaths associated with AMR, translating to an all-age rate of 2.4 deaths per 100,000 population [17]. Additionally, the associated burden in terms of DALYs was substantial, with 2,086,000 DALYs linked to AMR and an all-age rate of 189.7 DALYs per 100,000 population. When focusing on deaths directly attributable to AMR, diarrheal diseases caused approximately 6,280 deaths, with an all-age mortality rate of 0.6 deaths per 100,000 population [17]. The attributable DALYs burden was also notable, reaching 489,000, equivalent to 44.5 DALYs per 100,000 population [17]. These figures highlight the critical impact of AMR on health outcomes related to diarrheal diseases in the WHO African region.

Childhood diarrheal diseases strain African healthcare systems, further straining already fragile infrastructures [2, 18]. Healthcare providers and resources are overwhelmed by cases in hospitals and clinics with the demand for specialized care and medical supplies surpassing supply capacities. This also imposes significant economic costs on healthcare systems and families, particularly in resource-limited settings in Africa. Several studies indicate a considerable financial strain on the healthcare system due to childhood diarrheal diseases, posing a significant risk of impoverishment to households, as many households are pushed below the national poverty line while seeking treatment [18–20]. A systematic literature review identified 25 articles from 20 low- and middle-income countries including Tanzania, Malawi, Rwanda, Ethiopia, and Kenya that reported cost estimates for diarrhea illness and treatment in children under the age of five of which the average cost of illness was $36.56 per outpatient episode and $159.90 for inpatient episodes, with direct medical costs contributing a large proportion of the total direct costs [21]. These direct costs also strain the limited healthcare budgets in African countries as the perpetual cycle of illness and treatment perpetuates a cycle of expenditure, diverting funds that could be allocated to other pressing health priorities. These frequent episodes of diarrhea can cause stunting, malnourishment, and delayed brain development in later life, leading to significant productivity losses, diminishing human capital and hindering socioeconomic development [22].

## Zinc and ORS: essential components of diarrheal treatment

Zinc supplementation has been shown to reduce the duration and severity of diarrhea in children, and to prevent subsequent episodes [23]. Zinc is important for cellular growth, cellular differentiation and metabolism and deficiency limits childhood growth and decreases resistance to infections. Although severe zinc deficiency is rare in humans, mild to moderate deficiency may be common worldwide [3]. There are several mechanisms of action of zinc on acute diarrhea, some of which are specific to the gastrointestinal system as it restores mucosal barrier integrity and enterocyte brush-border enzyme activity, promotes the production of antibodies and circulating lymphocytes against intestinal pathogens, and has a direct effect on ion channels, acting as a potassium channel blocker of adenosine 3–5-cyclic monophosphate-mediated chlorine secretion [24]. A meta-analysis showed a significant reduction in diarrhea duration following zinc supplementation (95% CI − 25.06 to − 3.89; *P* = 0.007) among included studies, reinforcing its therapeutic role in managing childhood diarrhea [25]. A Cochrane review revealed that on average, children receiving zinc supplementation had a shorter duration of diarrhea with a mean difference of approximately 13 h (95% CI − 20.52 to − 6.31) [24]. While a subgroup analysis showed that this reduction was significant among children older than six months (RR − 0.32; 95% CI − 0.58 to − 0.06), there was no significant effect with zinc supplementation among children younger than six months [24]. These results coupled with high heterogeneity across the included studies demonstrated moderate evidence supporting the effectiveness of zinc supplementation in treating childhood diarrhea.

On the other hand, ORS replaces fluid and electrolytes and decreases luminal volume [26]. In many contexts, ORS is often used interchangeably with low-osmolarity oral rehydration solution (LOORS). However, while both are designed to treat dehydration and reduce the dependence on antibiotics, LOORS is a more specific formulation compared to standard ORS [27]. LOORS, having lower osmolarity (245 mOsm/L) than plasma (290 mMol/L), facilitates fast absorption of sodium and water and the active absorption of glucose and amino acids [28]. A systematic review and fixed effect meta-analysis showed a 69% (95% CI 51–80%) pooled relative reduction in diarrhea mortality among children aged 5 years and below treated with ORS [29]. The review further estimated that ORS may reduce diarrhea mortality by up to 93% [29]. In a randomized controlled trial, children aged 3–59 months treated with a combination of zinc and ORS had a shorter duration (95% CI 0.65–0.90) and lower incidence of diarrhea (CI 0.76–0.96) than children treated with ORS alone [30]. This demonstrates that while there is moderate evidence supporting the effectiveness of zinc supplementation, combining zinc and ORS can have more effect on the treatment of diarrheal diseases.

The combination of zinc and ORS also proves to be cost-effective. Zinc and ORS are relatively low-cost compared to traditional medical treatments and hospitalizations with the soaring costs of antibiotics, and lead to significant healthcare cost savings [31, 32]. Cost effectiveness analyses reveals that zinc supplementation among children aged one to five years is a highly cost-effective intervention and would avert about avert 1.423 DALYs per 100 households and year in developing countries. By preventing severe illness and complications, these interventions avert the burden of disability and premature mortality associated with diarrheal illnesses and ensure gains in other health indicators including quality-adjusted life years [33, 34]. Additionally, as a long-term implication, enhanced access to zinc and ORS can contribute to the reduction of AMR which is a global public health threat. By effectively treating diarrhea with these interventions, there is less reliance on antibiotics, which helps mitigate the emergence of antimicrobial-resistant strains of diarrhea-causing organisms [35, 36].

Albeit, there are several challenges in accessing these essential components for diarrheal treatment in Africa including but not limited to limited availability, affordability, supply chain challenges and lack of awareness [37]. Supply chain insufficiencies such as stockouts, logistical issues, and inadequate infrastructure causes for the irregular availability of these commodities. However, in cases where these commodities are available, the utilization is usually low across several countries which can be attributed to low level of advocacy on the importance of these commodities by the governments [1, 4, 38–41]. For many African families, cost continues to be a major obstacle to getting access to these commodities [37]. Out-of-pocket costs for impoverished households can still be prohibitive, even with efforts to subsidize the cost of these interventions. When families must choose between meeting other critical needs and buying zinc and ORS due to limited financial resources and competing health priorities, treatment for childhood diarrhea may be delayed or performed subpar [37]. More so, access to these commodities is hindered by a low awareness of these essential components among caregivers and healthcare providers [42, 43]. These interventions’ benefits and use may be unknown to many caregivers and healthcare providers. Therefore, zinc and ORS may be underutilized or improperly administered, reducing their effectiveness in treating childhood diarrhea. Notwithstanding the challenges of accessing zinc and ORS, there have been tremendous efforts to aid this situation.

## Empirical evidences and successful stories

Following the launch of the global diarrheal control program by the World Health Organization in 1978, several regions and countries across Africa have recorded significant changes resulting from the use of ORS for the treatment and prevention of diarrheal-related dehydration [44]. In areas where routine zinc supplementation was offered in a community-based program, diarrhea hospitalizations were decreased by 23%, with a 46% reduction on all-cause mortality [45]. In Nigeria, Uganda, and Kenya, within the time frame of 2012 to 2016, the Clinton Health Access Initiative (CHAI) implemented an access program that engaged health care providers, individual groups, and the government [46, 47]. Although the CHAI program did not have as much impact on zinc, the baseline range of the ORS coverage in these African countries rose from 38 to 44% [47]. The impact of the program proved to be significant as it heightened the combined ORS and zinc coverage levels across these countries: 30% coverage increase in eight different Nigerian states, 15% in Kenya and 30% in Uganda [47].

Following the efforts and meaningful partnerships between international health organizations like the Nutrition International and the Wellbeing Foundation Africa, several countries in Africa have also subscribed to the mission of increasing access to zinc and ORS [48]. Furthermore, the partnership program implemented by Nutrition international and the Wellbeing Foundation Africa, in Nigeria, recorded a completion rate of 2262, going beyond the 1,500 health workers initially targeted to be trained with the projected completion rate of 195.60% [48]. Further efforts towards this cause have led to the development of a formative research tool for the development of locally appropriate messages for the promotion of ORS and zinc [49]. Significant impacts have been recorded in the African region towards improving access to zinc and ORS. The strategy applied in these regions has been marked as being similar to the Diarrheal Alleviation through Zinc Therapy (DAZT) program implemented in India that increased the use of ORS and zinc from 15 to 40% and 2 to 18% respectively, in the Gujarat district [50]. This success is also consistent with the expectations of the effectiveness studies carried out in Bangladesh and India [30, 51, 52]. This calls for continued actions to improve child health.

## Strategies to strengthen access to zinc and ORS

Improved access to zinc supplementation and ORS holds significant promise in reducing childhood diarrhea morbidity and mortality [53]. Zinc plays a crucial role in reducing the severity and duration of diarrhea episodes, while ORS helps in preventing dehydration, a major complication of diarrheal illnesses [54]. To ensure better health outcomes, there is a need for proactive and continued actions to strengthen the access to zinc and ORS for childhood diarrhea treatment. First, the inclusion of zinc supplements and ORS in a country’s essential drugs/medicine list is a critical public health strategy, especially in regions where diarrheal diseases are prevalent. Many countries have not updated their Essential Medicines List (EML) to incorporate zinc and ORS co-packs according to the WHO recommendations, and to register these commodities as an ‘over the counter’ (OTC) medicine [55]. So far, 14 countries have ORS only in their EML, 24 countries have ORS and zinc separately, and seven countries have co-packs in their EML as of September 2024 [55]. Considering that both zinc supplements and ORS are relatively inexpensive, by including them in EML, governments can prioritize their procurement and distribution, ensuring cost-effective interventions for a widespread health issue and contribute to reducing child mortality and improving overall child health.

Second, addressing supply chain bottlenecks is fundamental to ensure the consistent availability and distribution of zinc and ORS across Africa. This entails strengthening procurement systems, optimizing inventory management, and establishing reliable distribution networks to facilitate the timely delivery of supplies to healthcare facilities at all levels. Collaborative partnerships between governments, international organizations, pharmaceutical companies, and non-governmental organizations can help streamline supply chain operations. These actions however must be backed up by robust monitoring and evaluation mechanisms, which is essential to track progress, identify gaps, and inform evidence-based decision-making in efforts to strengthen access to zinc and ORS. Routine data collection and analysis should be conducted to monitor the availability, distribution, and utilization of diarrhea treatment interventions, as well as to assess health outcomes and program effectiveness at health facility levels. These actions will ultimately lead to a management of commodities stock and mitigate stockouts.

Third, ensuring affordability of zinc and ORS is critical to overcoming barriers to access. Governments and stakeholders should explore strategies to reduce out-of-pocket costs for caregivers, such as subsidizing the price of zinc and ORS through public health financing mechanisms or health insurance schemes. This can draw on the International Zinc Procurement Fund (ZPF) recommended by Fisher-Walker and colleagues to ensure the inclusion of zinc in countries’ routine health budget [56]. Additionally, leveraging partnerships with pharmaceutical manufacturers to negotiate competitive pricing agreements can help lower the cost of procurement and improve affordability for healthcare systems. While national diarrheal control programs often suffer from underfunding, increasing the fiscal space for health in countries’ annual budget will ensure that more funds can be allocated to strengthen efforts aimed at reducing the burden of diarrheal diseases.

Fourth, considering that childhood illnesses are usually interrelated, zinc and ORS access should be strengthened in existing Integrated Management of Childhood Illnesses (IMCI) strategies. IMCI implementation has been effective in ensuring cost-saving for health systems in Africa as it provides unified and holistic healthcare for children under five and promotes efficient use of limited health resources [57, 58]. Thus, the promotion of zinc and ORS in IMCI will ensure better access to these commodities. This can also be achieved through targeted training on IMCI for frontline health workers, including nurses, midwives, community health workers, and pharmacists, equipping them with the necessary competencies to diagnose and treat diarrhea effectively. This strategy applied in Nigeria, Uganda, and Kenya by CHAI, Nutrition International and the Wellbeing Foundation Africa benefited health workers, under-five children, and the health systems in these countries to reduce diarrheal disease burden [46–48].

Fifth, community engagement and behavioral change communication are crucial to improving Zinc and ORS access for childhood diarrhea. Caregiver education should emphasize the benefits of the use of these commodities and the urgency of treating childhood diarrhea. While traditional beliefs about diarrhea treatment may hinder the adoption of ORS and zinc supplementation, culturally appropriate communication channels like community meetings, radio, and mobile messaging platforms can spread key messages and encourage adoption in various contexts. The successes of the programs implemented by CHAI, Nutrition International and the Wellbeing Foundation Africa across Nigeria, Uganda, and Kenya were attainable through working with religious and traditional leaders, community groups, ward development committees, and the health ministries to co-create strategies [46–48]. This strategy can be applied in other settings given a context-specific design. Lastly, collaboration with public facilities, private healthcare practitioners, and community-based organizations is vital to ensure equitable access to these commodities for childhood diarrhea treatment across all segments of the population.

## Conclusions

Zinc supplementation and ORS are cornerstone interventions in the management of childhood diarrhea as they help to rehydrate children by replacing lost fluids and electrolytes, while reducing the severity and duration of diarrhea episodes. Thus, it is necessary to prevent future occurrences, and improve overall health outcomes. Access to these essential commodities remains a critical challenge in the effective management of childhood diarrhea in Africa. Improving access to zinc supplementation and ORS offers a multifaceted approach to addressing childhood diarrhea, with implications for health, economics, and antimicrobial stewardship. Stakeholders from communities, health sector, and education must prioritize and invest in scaling up access to these essential interventions to maximize their impact on child health and development. By implementing key strategies such as addressing affordability of these commodities, training and capacity building, supply chain strengthening, community engagement, and monitoring and evaluation, significant progress can be made in overcoming barriers and improving health outcomes for children. Moreover, by promoting these commodities as first line treatment for childhood diarrhea and prioritizing these interventions in clinical guidelines and public health programs, they can be widely adopted and effectively utilized across African countries. Sustainable investment in strengthening healthcare systems and evidence-based interventions are essential to ensure that all children have equitable access to life-saving diarrhea treatment services, ultimately contributing to the reduction of childhood mortality and the attainment of global health goals in Africa.

## Data Availability

The dataset supporting this article’s conclusions are included.

## Abbreviations

AMR: Antimicrobial resistance
CHAI: Clinton health access initiative
DALY: Disability-adjusted life years
DAZL: Diarrheal alleviation through zinc therapy
EML: Essential medicines list
GBD: Global burden of disease
IHME: Institute for health metrics and evaluation
IMCI: Integrated management of childhood illnesses
LOORS: Low-osmolarity oral rehydration solution
ORS: Oral rehydration solution
OTC: Over the counter
UNICEF: United Nations children’s fund
WHO: World Health Organization
ZPF: Zinc procurement fund

## References

[1] Diarrhoea. UNICEF. 2024. https://data.unicef.org/topic/child-health/diarrhoeal-disease/. Accessed 13 February 2025

[2] Demissie GD, Yeshaw Y, Aleminew W, Akalu Y. Diarrhea and associated factors among under five children in sub-Saharan Africa: evidence from demographic and health surveys of 34 sub-Saharan countries. PLoS ONE. 2021;16(9):e0257522. 10.1371/journal.pone.0257522. 34543347 10.1371/journal.pone.0257522 PMC8452002

[3] Zinc supplementation in the management of diarrhoea. World Health Organization. 2023. https://www.who.int/tools/elena/interventions/zinc-diarrhoea. Accessed 10 February 2025

[4] Kassa SF, Alemu TG, Techane MA, Wubneh CA, Assimamaw NT, Belay GM, et al. The co-utilization of oral rehydration solution and zinc for treating diarrhea and its associated factors among under-five children in Ethiopia: further analysis of EDHS 2016. Patient Prefer Adherence. 2022;16:1713–21. 10.2147/PPA.S356557. 35903082 10.2147/PPA.S356557 PMC9314449

[5] Mohamed SOO, Alawad MOA, Ahmed AAM, Mahmoud AAA. Access to oral rehydration solution and zinc supplementation for treatment of childhood diarrhoeal diseases in Sudan. BMC Res Notes. 2020;13(1):427. 10.1186/s13104-020-05268-y. 32912300 10.1186/s13104-020-05268-y PMC7487982

[6] Patel AB, Mamtani M, Badhoniya N, Kulkarni H. What zinc supplementation does and does not achieve in diarrhea prevention: a systematic review and meta-analysis. BMC Infect Dis. 2011;11(1):122. 10.1186/1471-2334-11-122. 21569418 10.1186/1471-2334-11-122 PMC3115868

[7] Ahinkorah BO, Aboagye RG, Seidu AA, Frimpong JB, Cadri A, Afaya A, Hagan JE Jr, Yaya S. Prevalence and predictors of oral rehydration therapy, zinc, and other treatments for diarrhoea among children under-five in sub-Saharan Africa. PLoS ONE. 2022;17(10):e0275495. 10.1371/journal.pone.0275495. 36227873 10.1371/journal.pone.0275495 PMC9560133

[8] Owusu DN, Duah HO, Dwomoh D, Alhassan Y. Prevalence and determinants of diarrhoea and acute respiratory infections among children aged under five years in West Africa: evidence from demographic and health surveys. Int Health. 2024;16(1):97–106. 10.1093/inthealth/ihad046. 37387288 10.1093/inthealth/ihad046 PMC10759282

[9] Reiner RC, Graetz N, Casey DC, Troeger C, Garcia GM, Mosser JF, et al. Variation in Childhood diarrheal morbidity and mortality in Africa, 2000–2015. N Engl J Med. 2018;379(12):1128–38. 10.1056/NEJMoa1716766. 30231224 10.1056/NEJMoa1716766 PMC6078160

[10] GBD Compare. Institute for Health Metrics and Evaluation. 2025. https://vizhub.healthdata.org/gbd-compare/. Accessed 20 March 2025

[11] Mohamed AI, Abdilahi MM, Abdeeq BA, Mohamed J. Prevalence and associated factors of acute diarrhea among under-five children living in Hargeisa Internally Displaced Persons, Somaliland: a community-based cross-sectional study. Pan Afr Med J. 2024;47(10). 10.11604/pamj.2024.47.10.35958 10.11604/pamj.2024.47.10.35958 PMC10870163 38371646

[12] Bado AR, Susuman AS, Nebie EI. Trends and risk factors for childhood diarrhea in sub-Saharan countries (1990–2013): assessing the neighborhood inequalities. Glob Health Action. 2016;9(1):30166. 10.3402/gha.v9.30166. 27174860 10.3402/gha.v9.30166 PMC4865764

[13] Awuor AO, Ogwel B, Powell H, Verani JR, Sow SO, Hossain MJ, et al. Antibiotic-prescribing practices for management of childhood diarrhea in 3 Sub-Saharan African countries: findings from the vaccine impact on diarrhea in Africa (VIDA) Study, 2015–2018. Clinical Infectious Diseases. 2023;76(Supplement_1). 10.1093/cid/ciac980 10.1093/cid/ciac980 PMC10116514 37074427

[14] Neupane R, Bhathena M, Das G, Long E, Beard J, Solomon H, et al. Antibiotic resistance trends for common bacterial aetiologies of childhood diarrhoea in low- and middle-income countries: A systematic review. J Glob Health. 2023;13:04060. 10.7189/jogh.13.04060. 37475599 10.7189/jogh.13.04060 PMC10359834

[15] Rhee C, Aol G, Ouma A, Audi A, Muema S, Auko J, Omore R, Odongo G, Wiegand RE, Montgomery JM, Widdowson MA. Inappropriate use of antibiotics for childhood diarrhea case management—Kenya, 2009–2016. BMC Public Health. 2019;19:1–2. 10.1186/s12889-019-6771-8. 32326936 10.1186/s12889-019-6771-8 PMC6696675

[16] Gayawan E, Cameron E, Okitika T, Egbon OA, Gething P. A situational assessment of treatments received for childhood diarrhea in the Federal Republic of Nigeria. PLoS ONE. 2024;19(5):e0303963. 10.1371/journal.pone.0303963. 38776302 10.1371/journal.pone.0303963 PMC11111079

[17] Sartorius B, Gray AP, Weaver ND, Aguilar GR, Swetschinski LR, Ikuta KS, et al. The burden of bacterial antimicrobial resistance in the WHO African region in 2019: a cross-country systematic analysis. Lancet Glob Health. 2024;12(2):e201–16. 10.1016/S2214-109X(23)00539-9. 38134946 10.1016/S2214-109X(23)00539-9 PMC10805005

[18] Ngabo F, Mvundura M, Gazley L, Gatera M, Rugambwa C, Kayonga E, Tuyishime Y, Niyibaho J, Mwenda JM, Donnen P, Lepage P. The economic burden attributable to a child’s inpatient admission for diarrheal disease in Rwanda. PLoS ONE. 2016;11(2):e0149805. 10.1371/journal.pone.0149805. 26901113 10.1371/journal.pone.0149805 PMC4764684

[19] Hendrix N, Bar-Zeev N, Atherly D, Chikafa J, Mvula H, Wachepa R, Crampin AC, Mhango T, Mwansambo C, Heyderman RS, French N. The economic impact of childhood acute gastroenteritis on Malawian families and the healthcare system. BMJ Open. 2017;7(9):e017347. 10.1136/bmjopen-2017-017347. 28871025 10.1136/bmjopen-2017-017347 PMC5589001

[20] Niyibitegeka F, Riewpaiboon A, Youngkong S, Thavorncharoensap M. Economic burden of childhood diarrhea in Burundi. Glob Health Res Policy. 2021;6(1):13. 10.1186/s41256-021-00194-3. 33845920 10.1186/s41256-021-00194-3 PMC8042854

[21] Baral R, Nonvignon J, Debellut F, Agyemang SA, Clark A, Pecenka C. Cost of illness for childhood diarrhea in low- and middle-income countries: a systematic review of evidence and modelled estimates. BMC Public Health. 2020;20(1):619. 10.1186/s12889-020-08595-8. 32370763 10.1186/s12889-020-08595-8 PMC7201538

[22] Manetu WM, M.masi S, Recha CW. Diarrhea disease among children under 5 years of age: a global systematic review. Open J Epidemiol. 2021;11(3):207–21. 10.4236/ojepi.2021.113018.

[23] Saronga HP, Manji K, Liu E, Duggan CP, Menzies NA. Cost-effectiveness of zinc supplementation for prevention of childhood diarrhoea in Tanzania. Public Health Nutr. 2022;25(7):1–26. 10.1017/S1368980022000568. 10.1017/S1368980022000568 PMC9991691 35272738

[24] Lazzerini M, Wanzira H. Oral zinc for treating diarrhoea in children. Cochrane Database Syst Rev. 2016. 10.1002/14651858.CD005436.pub5. 27996088 10.1002/14651858.CD005436.pub5 PMC5450879

[25] Zou TT, Mou J, Zhan X. Zinc supplementation in acute diarrhea. Indian J Pediatr. 2015;82:415–20. 10.1007/s12098-014-1504-6. 24954892 10.1007/s12098-014-1504-6

[26] Digre P, Simpson E, Cali S, Lartey B, Moodley M, Diop N. Caregiver perceptions and utilization of oral rehydration solution and other treatments for diarrhea among young children in Burkina Faso. J Glob Health. 2016;6(2):1–11. 10.7189/jogh.06.020407. 10.7189/jogh.06.020407 PMC5032344 27699000

[27] Mohanty N, Thapa BR, Mathai J, Pai U, Mohanty N, Biradar V, et al. Low osmolarity oral rehydration salt solution (LORS) in management of dehydration in children. Indian Pediatr. 2021;58(3):266–72. 10.1007/s13312-021-2168-8. 33713063 10.1007/s13312-021-2168-8 PMC8005284

[28] Clinical management of acute diarrhoea: WHO/UNICEF joint statement. World Health Organization. 2004. https://iris.who.int/handle/10665/68627. Accessed 05 March 2025

[29] Munos MK, Walker CLF, Black RE. The effect of oral rehydration solution and recommended home fluids on diarrhoea mortality. Int J Epidemiol. 2010;39(Suppl 1):i75–87. 10.1093/ije/dyq025. 20348131 10.1093/ije/dyq025 PMC2845864

[30] Baqui AH, Black RE, Arifeen SE, Yunus M, Chakraborty J, Ahmed S, et al. Effect of zinc supplementation started during diarrhoea on morbidity and mortality in Bangladeshi children: community randomised trial. BMJ. 2002;325(7372):1059. 10.1136/bmj.325.7372.1059. 12424162 10.1136/bmj.325.7372.1059 PMC131175

[31] Brown KH, Hess SY, Vosti SA, Baker SK. Comparison of the estimated cost-effectiveness of preventive and therapeutic zinc supplementation strategies for reducing child morbidity and mortality in Sub-Saharan Africa. Food Nutr Bull. 2013;34(2):199–214. 10.1177/156482651303400209. 23964393 10.1177/156482651303400209

[32] Fink G, Heitner J. Evaluating the cost-effectiveness of preventive zinc supplementation. BMC Public Health. 2014;14:1–10. 10.1186/1471-2458-14-852. 25128210 10.1186/1471-2458-14-852 PMC4143582

[33] Fischer-Walker CL, Walker N. The lives saved tool (LiST) as a model for diarrhea mortality reduction. BMC Med. 2014;12(1):70. 10.1186/1741-7015-12-70. 24779400 10.1186/1741-7015-12-70 PMC4234397

[34] Bhutta ZA, Das JK, Walker N, Rizvi A, Campbell H, Rudan I, et al. Interventions to address deaths from childhood pneumonia and diarrhoea equitably: what works and at what cost? The Lancet. 2013;381(9875):1417–29. 10.1016/S0140-6736(13)60648-0. 10.1016/S0140-6736(13)60648-0 23582723

[35] Behera P, Pradhan SK, Behera SM, Rao EV. Zinc/ORS Co-packaging: a step towards bridging the gap in preventable childhood diarrhoeal deaths in India. Indian J Commun Med. 2023;48(3):505–6. 10.4103/ijcm.ijcm_1006_22. 10.4103/ijcm.ijcm_1006_22 PMC10353675 37469909

[36] Penny ME. Zinc supplementation in public health. Ann Nutr Metab. 2013;62(Suppl. 1):31–42. 10.1159/000348263. 23689111 10.1159/000348263

[37] Fagbamigbe AF, Juma J, Kariuki J. Barriers to utilisation of oral rehydration solution and zinc in managing diarrhoea among under-5 children in Oyo State, Nigeria. BMJ Paediatr Open. 2022;6(1):e001450. 10.1136/bmjpo-2022-001450. 36053606 10.1136/bmjpo-2022-001450 PMC9226878

[38] Akinyemi AI, Fagbamigbe AF, Omoluabi E, Agunbiade OM, Adebayo SO. Diarrhoea management practices and child health outcomes in Nigeria: sub-national analysis. Adv Integr Med. 2018;5(1):15–22. 10.1016/j.aimed.2017.10.002.

[39] Fagbamigbe AF, Joseph J, Odongo AO, Mogere D, Kariuki J. Evaluation of the awareness and utilisation of oral rehydration salt and zinc in managing diarrhoea among under-five children in Oyo State, Nigeria. Int J Commun Med Pub Health. 2022;9(7):2785.

[40] Terefa DR, Shama AT. Predictors of under-five caregivers’ utilization of co-packaged zinc and oral rehydration salts for childhood diarrhea in East Wollega zone Western Ethiopia. Patient Prefer Adherence. 2023;17:913–26. 10.2147/PPA.S405054. 37016674 10.2147/PPA.S405054 PMC10066899

[41] Yeshaw Y, Worku MG, Tessema ZT, Teshale AB, Tesema GA. Zinc utilization and associated factors among under-five children with diarrhea in East Africa: a generalized linear mixed modeling. PLoS ONE. 2020;15(12):e0243245. 10.1371/journal.pone.0243245. 33264367 10.1371/journal.pone.0243245 PMC7710063

[42] Amu EO, Olatona FA, Adeyemi BO, Adegbilero-Iwari OE. Childhood diarrhoea in southwestern Nigeria: predictors of low osmolarity ORS and zinc use among mothers. J Taibah Univ Med Sci. 2022;17(6):1006–13. 10.1016/j.jtumed.2022.05.003. 36212577 10.1016/j.jtumed.2022.05.003 PMC9519615

[43] Uzoma OJ, Chiejina EN. Knowledge and barriers to use of low-osmolarity oral rehydration solution and zinc supplementation in the management of childhood diarrhea among primary health care providers in Imo State, Nigeria. Afr J Biol Med Res. 2021;4(3):79–91. 10.52589/AJBMR-BDDCN0IZ

[44] Nalin DR, Cash RA. 50 years of oral rehydration therapy: the solution is still simple. Lancet. 2018;392(10147):536–8. 10.1016/S0140-6736(18)31488-0. 30152375 10.1016/S0140-6736(18)31488-0

[45] Fischer-Walker CL, Black RE. Zinc supplementation for diarrhoea treatment. World Health Organization. 2014. https://www.who.int/tools/elena/commentary/zinc-diarrhoea. Accessed 18 October 2024

[46] Black RE. Progress in the use of ORS and zinc for the treatment of childhood diarrhea. J Global Health. 2019;9(1):010101. 10.7189/jogh.09.010101. 10.7189/09.010101 PMC6344068 30701067

[47] Schroder K, Battu A, Wentworth L, Houdek J, Fashanu C, Wiwa O, Kihoto R, Macharia G, Trikha N, Bahuguna P, Dabas H. Increasing coverage of pediatric diarrhea treatment in high-burden countries. J Global Health. 2019;9(1):0010503. 10.7189/jogh.09.010503. 10.7189/jogh.09.010503 PMC6513503 31131105

[48] Improving Diarrhea Treatment Outcome through the Use of Zinc and LO-ORS in Northern Nigeria The Wellbeing Foundation Africa. 2023. https://wbfafrica.org/improving-diarrhea-treatment-outcome-through-the-use-zinc-and-lo-ors-in-northern-nigeria-phase2/. Accessed 11 February 2025

[49] Nichter M, Acuin CS, Vargas A. Introducing zinc in a diarrhoeal control programme: guide to conducting formative research. World Health Organization. 2008. https://iris.who.int/handle/10665/43855. Accessed 23 February 2025

[50] Lamberti LM, Taneja S, Mazumder S, LeFevre A, Black RE, Walker CL. An external evaluation of the diarrhea alleviation through Zinc and ORS treatment (DAZT) program in Gujarat and Uttar Pradesh, India. J Global Health. 2015;5(2):020409. 10.7189/jogh.05.020409. 10.7189/jogh.05.020409 PMC4676588 26682045

[51] Bhandari N, Mazumder S, Taneja S, Dube B, Agarwal R, Mahalanabis D, et al. Effectiveness of zinc supplementation plus oral rehydration salts compared with oral rehydration salts alone as a treatment for acute diarrhea in a primary care setting: a cluster randomized trial. Pediatrics. 2008;121(5):1279–85. 10.1542/peds.2007-1939. 10.1542/peds.2007-1939 18450870

[52] Mazumder S, Taneja S, Bhandari N, Dube B, Agarwal R, Mahalanabis D, et al. Effectiveness of zinc supplementation plus oral rehydration salts for diarrhoea in infants aged less than 6 months in Haryana state. India Bull World Health Organ. 2010;88(10):754–60. 10.2471/BLT.10.075986. 20931060 10.2471/BLT.10.075986 PMC2947049

[53] Yakoob MY, Theodoratou E, Jabeen A, Imdad A, Eisele TP, Ferguson J, Jhass A, Rudan I, Campbell H, Black RE, Bhutta ZA. Preventive zinc supplementation in developing countries: impact on mortality and morbidity due to diarrhea, pneumonia and malaria. BMC Public Health. 2011;11:1–10. 10.1186/1471-2458-11-S3-S23. 21501441 10.1186/1471-2458-11-S3-S23 PMC3231897

[54] The treatment of diarrhoea : a manual for physicians and other senior health workers, 4th rev. World Health Organization. 2005. https://iris.who.int/handle/10665/43209. Accessed 12 February 2025

[55] ORS and Zinc Status Around the World. The ORS/Zinc Co-pack Alliance (ORSZCA). 2024. https://orszco-pack.org/orsz-status/. Accessed 5 December 2024

[56] Fischer-Walker CL, Fontaine O, Young MW, Black RE. Zinc and low osmolarity oral rehydration salts for diarrhoea: a renewed call to action. Bull World Health Organ. 2009;87(10):780–6. 10.2471/BLT.08.058990. 19876545 10.2471/BLT.08.058990 PMC2755312

[57] Boschi-Pinto C, Labadie G, Dilip TR, Oliphant N, Dalglish SL, Aboubaker S, Agbodjan-Prince OA, Desta T, Habimana P, Butron-Riveros B, Al-Raiby J. Global implementation survey of Integrated management of childhood illness (IMCI): 20 years on. BMJ Open. 2018;8(7):e019079. 10.1136/bmjopen-2017-019079. 30061428 10.1136/bmjopen-2017-019079 PMC6067364

[58] Jacobs M, Merson M. Introductory commentary: a strategic review of options for building on lessons learnt from IMCI and iCCM. BMJ. 2018;362. 10.1136/bmj.k3013 10.1136/bmj.k3013 PMC6081995 30061381

